# Genome re-sequencing and reannotation of the *Escherichia coli* ER2566 strain and transcriptome sequencing under overexpression conditions

**DOI:** 10.1186/s12864-020-06818-1

**Published:** 2020-06-16

**Authors:** Lizhi Zhou, Hai Yu, Kaihang Wang, Tingting Chen, Yue Ma, Yang Huang, Jiajia Li, Liqin Liu, Yuqian Li, Zhibo Kong, Qingbing Zheng, Yingbin Wang, Ying Gu, Ningshao Xia, Shaowei Li

**Affiliations:** 1 State Key Laboratory of Molecular Vaccinology and Molecular Diagnostics, School of Public Health, Xiamen University, Xiamen, 361102 Fujian China; 2 National Institute of Diagnostics and Vaccine Development in Infectious Disease, School of Life Sciences, Xiamen University, Xiamen, 361102 Fujian China

**Keywords:** *Escherichia coli* ER2566, Genome reannotation, Transcriptome sequencing, Engineer bacteria

## Abstract

**Background:**

The *Escherichia coli* ER2566 strain (NC_CP014268.2) was developed as a BL21 (DE3) derivative strain and had been widely used in recombinant protein expression. However, like many other current RefSeq annotations, the annotation of the ER2566 strain was incomplete, with missing gene names and miscellaneous RNAs, as well as uncorrected annotations of some pseudogenes. Here, we performed a systematic reannotation of the ER2566 genome by combining multiple annotation tools with manual revision to provide a comprehensive understanding of the *E. coli* ER2566 strain, and used high-throughput sequencing to explore how the strain adapted under external pressure.

**Results:**

The reannotation included noteworthy corrections to all protein-coding genes, led to the exclusion of 190 hypothetical genes or pseudogenes, and resulted in the addition of 237 coding sequences and 230 miscellaneous noncoding RNAs and 2 tRNAs. In addition, we further manually examined all 194 pseudogenes in the Ref-seq annotation and directly identified 123 (63%) as coding genes. We then used whole-genome sequencing and high-throughput RNA sequencing to assess mutational adaptations under consecutive subculture or overexpression burden. Whereas no mutations were detected in response to consecutive subculture, overexpression of the human papillomavirus 16 type capsid led to the identification of a mutation (position 1,094,824 within the 3′ non-coding region) positioned 19-bp away from the *lac*I gene in the transcribed RNA, which was not detected at the genomic level by Sanger sequencing.

**Conclusion:**

The ER2566 strain was used by both the general scientific community and the biotechnology industry. Reannotation of the *E. coli* ER2566 strain not only improved the RefSeq data but uncovered a key site that might be involved in the transcription and translation of genes encoding the lactose operon repressor. We proposed that our pipeline might offer a universal method for the reannotation of other bacterial genomes with high speed and accuracy. This study might facilitate a better understanding of gene function for the ER2566 strain under external burden and provided more clues to engineer bacteria for biotechnological applications.

## Background

The *Escherichia coli* expression system is one of the most well-characterized classical expression systems for recombinant protein expression in biological science. *E. coli* offers clear advantages over other expression systems, including a clear genetic background, fast breeding, low cost, high cell density cultures, and high protein expression levels [1–3]. Indeed, more than 60% of recombinant proteins and nearly 30% of approved recombinant therapeutic proteins are produced using *E. coli* expression systems [4]. The *E. coli* ER2566 strain is a common laboratory tool that takes advantage of the expression and growth properties of the B line strain [5]. In 2016, the first complete genome of ER2566 (C2566, NC_CP014268.2) competent cells was sequenced by New England Biolabs and deposited into GenBank [6], with automatic annotation by the NCBI prokaryotic genome annotation pipeline (PGAP) [7].

Along with the rapid development of biological laboratory techniques, there has been a significant advance in sequencing technologies. However, this has not been matched by the production of better sequences; rather, sequencing advancements have led to the deposition of an increased number of “draft” bacterial genomes into public databases [8], which tend to be incomplete and fragmented. In addition, the massive amounts of genomic data generated by next-generation sequencing platforms has also increased the probability of errors in genome annotations, since most genomes are annotated automatically and not subjected to any manual review. High-quality annotations of bacterial genomes are critical to understanding biological processes and enhancing these genomes has become a major task in the post-genomic era. Therefore, the reannotation of previously published genomes with manual reviewing is necessary to improve databases and supply accurate information [9]. Although numerous tools have been used to identify relevant genes, gene prediction is still imperfect. In addition, some genuine genes are missed by gene finder tools because the algorithms are directed to maintain a balance between specificity and sensitivity to avoid false-positive predictions. Thus, the use of multiple ab initio gene finders along with BLAST searching will help to identify genes correctly and lead to more accurate annotations [10, 11].

Next-generation sequencing (NGS) is a cost-effective tool for the study of gene function and experimental bacterial evolution. Indeed, NGS technology was used by Luhachack and colleagues to successfully identify the function of the transcription factor *Ycj*W as a regulator of the complex interaction between carbohydrate metabolism and H2S production in bacteria [12], and whole-genome re-sequencing in *E. coli* by Herring et al. led to the identification of mutations that conveyed a selective growth advantage during adaptation to a glycerol-based growth medium [13]. Even though the ER2566 engineering strain is widely used for the preparation of virus-like particles [14], and bacterial surface display [15], among other functions, exploring bacterial adaptive evolution of the ER2566 strain under various external pressures through NGS remains difficult and inconclusive because of limitations in the annotations of the strain [13].

In this study, we employed a series of automated annotations and combined this with manual inspections with high-throughput analyses to reannotate the ER2566 genome. As a result of our analysis, we further propose a universal reannotation pipeline for other bacterial genomes that can be undertaken with high speed and accuracy. Our reannotation eliminated 190 hypothetical genes and pseudogenes from the RefSeq annotation, and now includes an additional 237 coding sequences with definitive gene names and functions. The number of miscellaneous noncoding RNAs was also increased from 15 to 245. Subsequently, we applied whole-genome sequencing and RNA-sequencing to assess for mutational adaptations that occur following continuous subculture pressure or overexpression burden. We detected a mutation located within the 3′ non-coding region (1,094,824 position) 19-bp away from the *lac*I gene at the RNA level, which may be involved in the transcription and translation of genes encoding the lactose operon repressor. Our reannotation and sequencing results will provide a better understanding of some of the biological processes of the ER2566 strain, and may offer insight into future biotechnological applications in bacterial engineering.

## Results & discussions

**Fig. 1:**
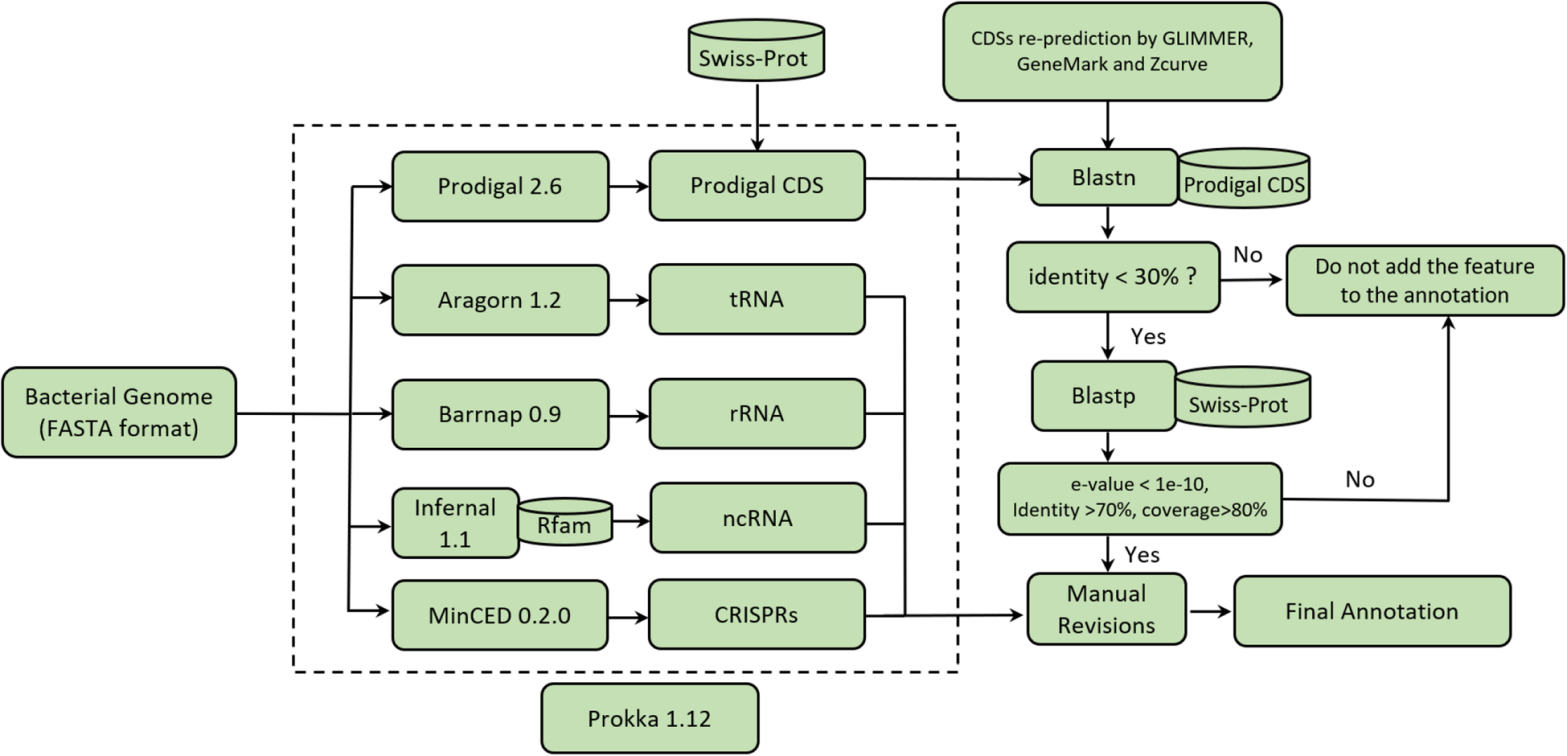
Flowchart depicting the pipeline and methods used for bacterial genome reannotation of the *E. coli* strain ER2566

The process of genome reannotation combined with detailed manual reviewing encompasses the re-identification and labeling of characteristic features of a sequenced genome, and is a process that has been performed extensively for numerous organisms, including bacteria [16, 17]. Unlike the human genome, which is about 1.3% protein coding, 90% of the bacterial genome codes for proteins, with only short intergenic stretches [18]. Precise genomic annotation is thus fundamental to the further interpretation of the biochemical and physiological characteristics of organisms, to provide detailed information on protein coding sequences, pseudogenes, non-coding RNAs, repeat sequences and various other genomic data [19]. In this study, we reannotated the genome of the *E. coli* strain ER2566 through a reannotation pipeline, as illustrated in Fig. 1, with high speed and accuracy.

We employed a series of automated annotation tools combined with manual inspection to reannotate the ER2566 genome (Fig. S1). In our pipeline, the automation part using Prokka combined with others gene finders (GLIMMER, Zcurve and GeneMark) could finish a complete bacterial genome annotation in about 30 min. Compared with some online tools, this pipeline showed higher speed and accuracy. For example, NCBI provides a Prokaryotic Genomes Annotation Pipeline service via email, with a turn-around time of several days [7]. RAST is another web server for annotating bacterial and archaeal genomes that provides results within 1 day [20]. Some local stand-alone annotation tools, such as RATT [21], Rapid Annotation Transfer Tool, can transfer annotations from a high-quality reference to a new genome on the basis of conserved synteny. However, due to the limitation of its algorithm, RATT cannot effectively identify pseudogenes, indels, etc. Besides, numerous automated tools have been developed for genome annotation, including Mypro [22], MAKER [23], BlastLKOLA [24] and so on. To avoid false-positive predictions, the algorithms of these annotation tools are designed to balance specificity and sensitivity of their results [25]. In contrast, combination of multiple ab initio gene finders combined with BLAST searching and manual inspection will help to confirm identified genes and generate more accurate annotations.

### Improvement in coding sequences (CDS)

For the systematic reannotation of the CDS, the prediction and identification of coding genes occurred in two stages (Fig. S2). In the first stage, Prodigal software was used to predict a total of 4180 CDSs on the complete ER2566 genome deposited in GenBank (accession number NC_CP014268.2). Using sequence alignment to the Swiss-Prot database [26] by Blastp [27], with a threshold e-value of < 10^− 6^, all CDSs were annotated to provide accurate information regarding the sequences and functions of the enrolled proteins. A total of 4023 (96.2%) of the 4180 CDSs were annotated as protein-coding genes, with the remaining CDSs (136 CDSs; 3.3%) marked as hypothetical genes, with no registration in the Swiss-Prot database, and 21 CDSs (0.5%) marked as pseudogenes by manual inspection. To improve upon this prediction, three other well-established gene finders-GLIMMER, Zcurve and GeneMark-were independently used, identifying 4231, 4287, and 4213 CDSs, respectively. These putative CDS sets were subsequently filtered by Blastn against the first Prodigal-predicted CDS set. Overall, an additional 428 CDSs were found: 194 CDSs were identified using GLIMMER, 123 using GeneMark, and 201 using ZCURVE. These additional 428 CDSs were then searched against the Swiss-Prot database by Blastp with a stricter threshold e-value < 10^− 10^, coverage > 80%, and an identity > 70%. This filtered out 402 of these additional CDSs, resulting in an additional 43 genes. This led to a total of 4066 protein-coding CDSs (4023 + 43) included in the reannotation of the ER2566 genome, along with 136 CDSs for hypothetical genes.

**Fig. 2:**
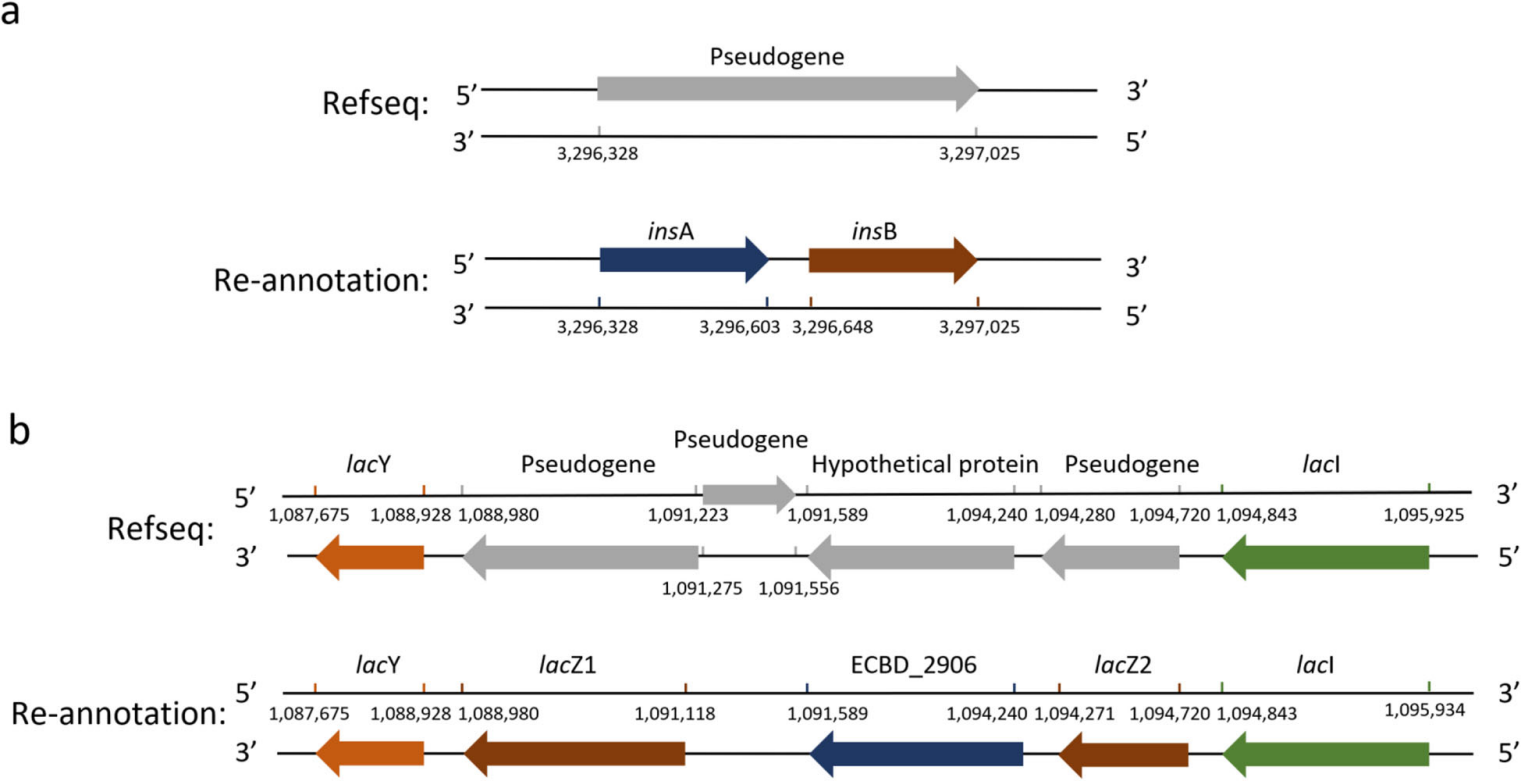
Examples of the differences between the original RefSeq annotation and our reannotation. **a** In the reannotation, one pseudogene (RS16270) was identified as two genes, *ins*A and *ins*B, which show strong homology to the insertion element protein, IS1. **b** In the reannotation, two pseudogenes were re-identified as two genes (*lac*Z1 and *lac*Z2), whereas the hypothetical protein was reannotated and shown to be highly homologous with the DNA-directed RNA polymerase gene ECBD_2906 from *E. coli* strain BL21-DE3

Due to trimming or splitting, genes with real function can often be incorrectly assigned as pseudogenes through protein homology alignment. In our reannotation, we manually reviewed and analyzed 194 pseudogenes from the RefSeq database annotation. In total, 123 of the 194 pseudogenes were directly identified as coding genes and are now found in the reannotated list. While the remaining pseudogenes without any function had been retained. These newly identified protein-coding genes included 34 mobile genetic elements that encode transposases and had been considered to be important in evolution as a common type of genetic change. For instance, a genomic positive strand region (3,296,328 - 3,297,025 bp), previously annotated as a pseudogene without function, was identified to harbor two genes, insA and insB, which are homologues of the insertion element protein, IS1, and related to DNA binding and transposase activity (Fig. 2a) [28]. In addition, two annotated pseudogenes (C2566-RS05300 and C2566_RS05310) and one hypothetical protein (C2566_RS22600) in the RefSeq annotation (range 1,088,980-1,094,720) were reannotated as three new genes (*lac*Z1, *lac*Z2, and ECBD_2906, respectively), and one related pseudogene, C2566_RS05305, was removed; these changes are consistent with previous results [5] (Fig. 2b). These three new genes are flanked by the lactose permease gene *lac*Y upstream and the lactose operon repressor gene *lac*I downstream, both of which are essential to the lac operon system. This reannotation had uncovered genes related to the transcription and translation of genes encoding the lactose operon repressor in ER2566 strain.

**Fig. 3:**
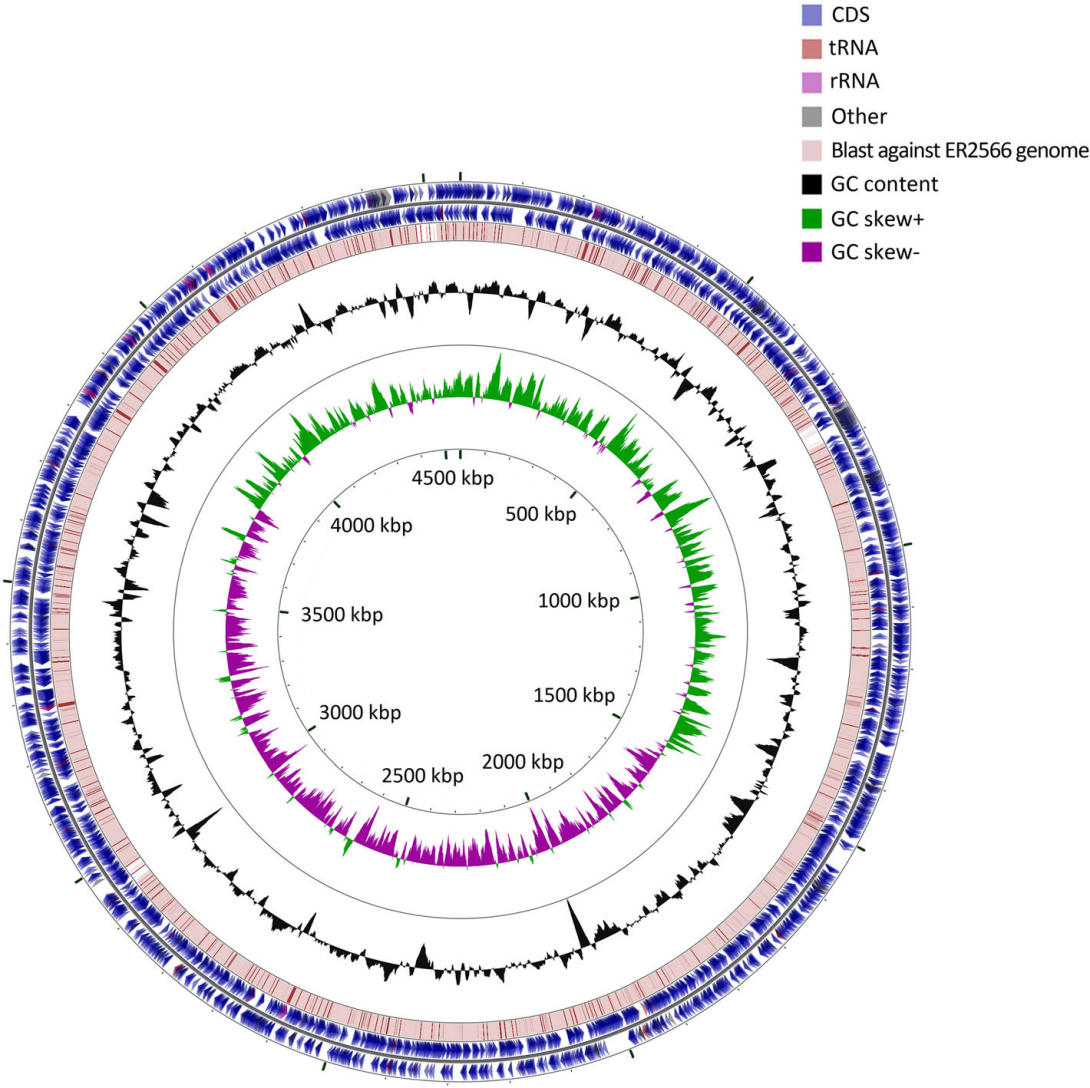
Comparison between BL21(DE3) genome and ER2566 genome. Viewing from outside to inside rings, the outermost two rings, respectively representing plus-strand and minus-strand, show features extracted from the BL21(DE3) genome GenBank file (GenBank: CP001509.3); the next ring shows the positions of BLAST hits between the BL21(DE3) genome and the ER2566 genome detected by Blastn. The height of each line in the third ring showing BLAST results is proportional to the percent identity of the hit, and overlapping hits renders as darker lines. The next two rings show GC content and GC skew

In comparing to RefSeq annotation, a total of 190 protein-coding CDSs were removed, as they were identified as either hypothetical proteins or pseudogenes, with most of them having no assigned function (Additional file 1). Meanwhile, 237 new CDSs were added (Additional file 2). The complete reannotation list can be found in Additional file 3. ER2566 strain is a BL21/K-12 hybrid strain, where about 6% sequence of K-12 strain replaces about 7% sequence in BL21(DE3) genome. The genome alignment of BL21 and ER2566 demonstrates a high degree of consistency [29] (Fig. 3). The recent version of BL21(DE3) annotation contains 4197 CDSs, in which 3873 (92.3%) CDSs were identified in ER2566 annotation as identical gene symbols or alias as well. The unidentical 7.7% CDSs annotated in BL21 genome as compared to ER2566 is comprised of ~ 7% sequence corresponding to the hybrid part in ER2566 and other CDSs that does not have an official gene name (Additional file 4). Overall, we determined 4197 protein-coding genes for the ER2566 genome, including 136 CDSs labeled as hypothetical proteins and 4061 CDSs, which 3873 CDSs identical to BL21(DE3) that account for about 99% of total 3903 CDSs (4197*93%) equivalent to BL21(DE3) CDSs within ER2566 genome. This reannotation effectively eliminated the possibility of false interpretations introduced by the original annotation and provides a more integral view of the regulatory networks in ER2566 strain (Table 1).

**Table 1:** Overview of the differences between the original annotation, the reannotation and BL21(DE3) annotation

	Original annotation (NZ_CP014268.2)	Reannotation	BL21(DE3)
Genome length	4,478,958 bp		4,558,953 bp
Plasmids	None		
G + C%	50.81%		50.83%
Genes (total)	4364	4627	4440
Protein_coding genes	4054	4202	4197
Pseudo Genes	194	71	70
tRNAs	85	87	85
rRNAs	22	22	22
Miscellaneous RNAs^a^	9	245	66
Backbone genes	4170 (4054 protein-coding genes,85 tRNA genes,22rRNAs and 9 misc. RNAs)	4556 (4202 protein-coding genes,87 tRNA genes,22rRNAs and 245 misc. RNAs)	4370 (4197 protein-coding genes, 85 tRNA genes,22rRNAs and 66 misc. RNAs)

^a^: The concept of miscellaneous RNA includes ncRNA, tmRNA and all other ncRNAs

Integrated proteogenomics search database (iPtgxDB) is widely used to provide protein expression evidence and could confirm the validity of the annotation, which was used to identify the short protein-coding genes that have numerous functions [30]. Thus, the combination of transcriptome data and reannotation results was used to generate an integrated proteomics database, which provided an important optimal basis for genome-scale regulatory or metabolic predictions and comprehensive exploration on the genome information and underlying gene functions (Additional file 5).

### Miscellaneous RNAs improvement

Miscellaneous RNAs, such as transfer RNA (tRNA), ribosomal RNA (rRNA), and other non-coding RNAs (ncRNA), play pivotal biological roles in cellular activity. To date, about 119 RNAs molecules in the *E. coli* ER2566 strain have been identified, including 85 tRNAs, 22 rRNAs, and 12 ncRNAs [6]. However, almost all of these ncRNAs are missing from the original RefSeq annotation. We used Aragorn 1.2, Barrnap 0.9, and infernal 1.1 ncRNA finders independently to predict genes coding for tRNAs, rRNAs, and ncRNAs. Compared with the auto-annotation, 2 tRNAs and 230 ncRNAs are new, with most having functions in translation, DNA replication, and expression regulation (Additional file 6). In addition, most of the ncRNAs functions have been verified experimentally. For instance, about 94 of the nucleoid-associated ncRNAs molecules play key functions in DNA-RNA interactions [31]. Meanwhile, fragments per kilobase of transcript per million mapped reads (FPKM) value was used to quantify the transcription level of the newly added ncRNAs and to analyze the transcriptomics data of ER2566 under different induction conditions (Additional file 7). Quantitative analysis indicates that 85% (208/245) of newly added ncRNAs are detectable, while the other 15% (37/245) ncRNAs are undetectable possibly due to some specific functions requiring certain conditions. In summary, our reannotation introduced 230 new ncRNAs and 2 new tRNAs, with an overall tally of genes encoding for 87 tRNAs, 22 rRNAs and 245 ncRNAs.

### Variant calling of whole-genome re-sequencing under consecutive subculture

**Fig. 4:**
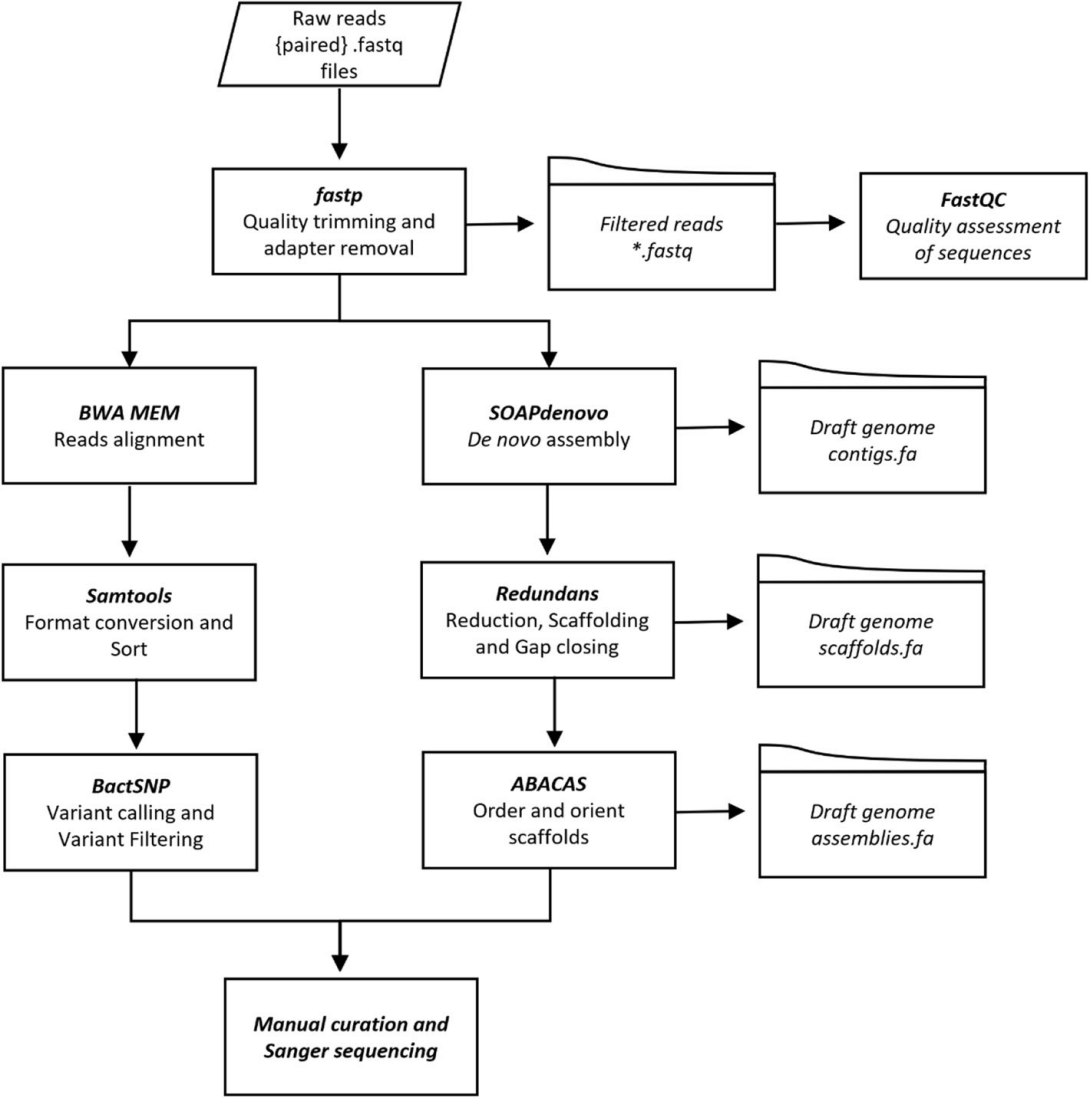
Flow-chart of variant calling, combining reads mapping and de novo assembly

Genomic variations in bacterial species usually reflect an evolutionary response that occurs under various external—usually unfavorable—environmental stressors. Thus, we next performed variant calling to identify any nucleotide-level differences (i.e., single nucleotide polymorphisms (SNPs), insertions and deletions (indels), and/or structural variations) in the ER2566 strain. There are two approaches for variant calling: by mapping reads against the reference genome directly or by assembling a de novo genome to compare against a reference genome. In most cases, mapping reads produces a better resolution for SNPs and indels than genome assembly, whereas the latter is optimal for identifying structural variants and regions with high divergence. Here, we used both methods to interrogate the ER2566 genome (Fig. 4).

First, we re-sequenced the whole genome of the ER2566 strain grown in our laboratory under consecutive subculturing. Two different-sized insert libraries (500 bp, 2000 bp) were built, and a total of 10.0 million paired-end reads of 90 bp in length were generated using an Illumina HiSeq 2000. The raw reads were mapped to the C2566 reference genome (NC_CP014268.2) with a good coverage depth (> 100-times). No SNP or indel was detected. A pipeline optimized for longer assembly was designed to accomplish the re-sequencing of our ER2566 strain. Various de novo assembly softwares were used to construct a confident and long (4,469,460-bp) scaffold, and assembly results for each step were assessed by alignment of final sequences back to the reference genome (Fig. S3). One inversion was found (Fig. S3c), which turned out to be a genome assembly issue caused by high repetition region and was corrected by subsequent Sanger sequencing. The technical difference between short-read sequencing and single molecule long-read sequencing may result in the generation of inverted region. Nevertheless, our pipeline, by the combination of read mapping, de novo assembly and Sanger confirmation, generated an intact ER2566 genome in our practice. The resequencing of ER2566 also suggested that the continuous subculture of *E. coli* ER2566 strain in our lab did not cause mutation in the genomic sequence.

### Mutation detections by RNA-sequencing under overexpression

**Fig. 5:**
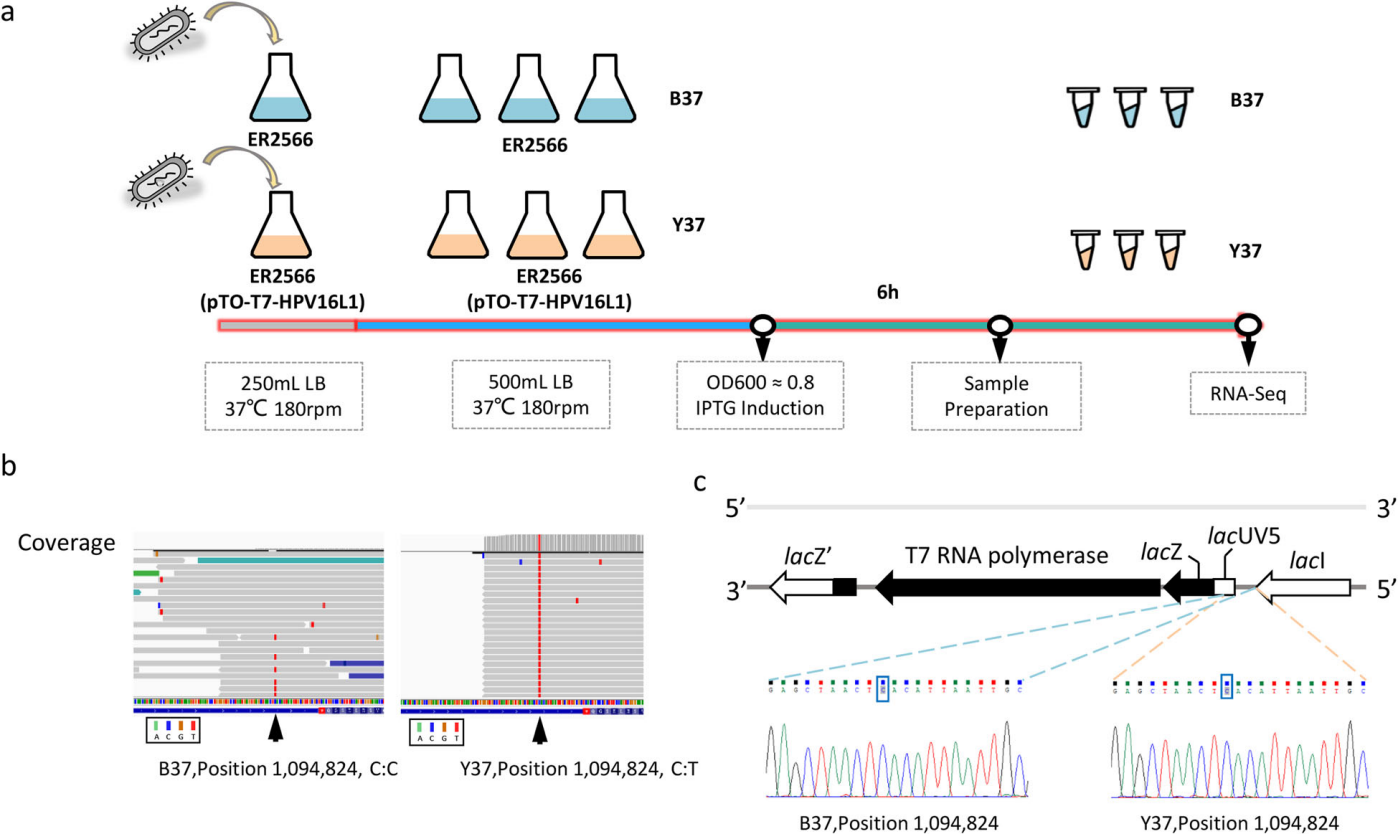
RNA-seq for variant calling under pressure from overexpression. a) The experimental design. Each group (B37, without plasmid; Y37, with pTO-T7 plasmid overexpressed) had three biological replicates. b) Visualization of BAM files of the B37 (left panel) and Y37 (right panel) in the Integrative Genomics Viewer. Based on the reannotation, one mutant was identified at position 1,094,824 C > T, located within the 3′ non-coding region of the transcription factor gene *lac*I. c) Mutation detected by Sanger sequencing of the B37 and Y37 genomic samples

RNA-seq is widely used in quantitative gene expression studies for the identification of non-annotated transcripts and polymorphisms, and for RNA editing in transcribed regions. Thus, to identify any variations in the ER2566 genome due to overexpression pressure, we used RNA-seq to analyze the transcriptomes of the ER2566 strain growing at 37 °C without plasmids (B37, three replicates) or overexpressing human papillomavirus 16 type capsid protein L1 via plasmid-based inducible expression (Y37, three replicates) (Fig. 5a). From a total of 75.4 million 125-bp paired-end reads, 73.9 million reads (98%) were mapped to the reference genome (NC_CP014268.2) in B37 (control) samples. Yet, for the Y37 (overexpressed) samples, among the 76. 0 million reads, only 29.6 million (39%) reads mapped to the reference genome, which was significantly lower than that for the B37 samples. The cause of lower mapping rates in Y37 samples was due to the large number of mRNAs transcribed by the engineered plasmids which is not related to genome sequence (Table 2).

**Table 2:** Statistical analysis of RNA-seq data

Sample	Run	Raw sequences reads	Unidentified reads^a^	HPV16L1 reads^b^	*E. coli* reads^b^
B37	1	22,781,394 (100%)	250,596 (1%)	0 (0%)	22,334,273 (99%)
	2	22,763,848 (100%)	318,694 (1%)	0 (0%)	22,312,577 (99%)
	3	29,884,996 (100%)	597,700 (2%)	0 (0%)	29,276,302 (98%)
Y37	1	28,292,578 (100%)	4,526,812 (16%)	12,731,660 (45%)	11,034,106 (39%)
	2	25,214,878 (100%)	4,286,528 (17%)	11,346,696 (45%)	9,581,654 (38%)
	3	22,521,416 (100%)	3,378,212 (15%)	10,134,638 (45%)	9,008,566 (40%)

^a^: Three biological replicates of each samples were analyzed. Unidentified RNA-seq reads could include unidentified nucleotides (Ns), short reads, low quality reads, unaligned recombinant protein reads and a larger number of mRNA from plasmids. ^b^: HPV16L1 reads and E. coli reads respectively represent Human papillomavirus and *Escherichia coli* organism reads

The variant detection analysis using BactSNP revealed one mutation in the Y37 samples (1,094,824 position; Fig. 5b), located in the non-coding region 19-bp downstream from the *lac*I gene. Interestingly, *lac*I is the highest transcribed gene in the Y37 samples by comparing the FPKM for all genes (Additional file 8). The analysis of transcriptome data indicated a mutation of C to T substitution at position 109,824 in the three replicates of Y37 samples, as confirmed by nearly 100% mutation rate in all observed reads (910,914 Out 911,345 reads). Furthermore, the mutation was found in three replicates of B37 samples as well, with a mutation rate up to 85% (655 Out 773 reads). Surprisingly, such mutation could not be detected in the bacterial genome by Sanger sequencing (Fig. 5c). The discrepancy between transcribed RNA and genome sequence may arise from the modification during RNA transcription instead of a sequencing error. The presumption is supported by the mutation position being located in a high-efficiency RNA methylation site, which is often accompanied with spontaneous deamination of 5-methylcytosine and consequently producing thymine in aqueous solution [32]. It is interesting to clarify whether the overexpression of lacI could lead to the increase of RNA methylation rate. The function of this site will be further investigated in our future work, using a combination of gene editing, RNA methylation analysis, and other relevant techniques.

## Conclusions

Here, we employed a series of automated annotation tools along with manual inspection to reannotate the ER2566 genome. The major updates include the noteworthy correction of all protein-coding genes, the exclusion of 190 CDSs from the Refseq annotation, and the addition of 237 new CDSs with definitive name or putative function. Moreover, there is an increase in the number of miscellaneous RNAs from 15 to 245. These new additions will help to provide a more informative profile of the ER2566 genome and provide a better base for exploring the molecular mechanisms of stress in response to changes in the bacterial cellular milieu. Nevertheless, this reannotation still has further room for improvement, with the continuing advancement of the algorithm, the accumulation of next-generation sequence data and proteomics data. We also carried out whole-genome sequencing and RNA-seq to detect sequence variants under different conditions of external pressure, and detected one mutation within the non-coding region of the *lac*I gene. However, this mutation was not detected at the genomic level by Sanger sequencing, which may indicate that this is an RNA modification related to the biological strain of overexpression pressure in ER2566.

The ER2566 strain is used widely within the scientific community, and our reannotation not only improved the characterization of the strain but uncovered a key site that might be involved in the transcription and translation of genes encoding the lactose operon repressor. Our reannotation pipeline with high speed and accuracy could thus be extrapolated for the reannotation of other bacterial genomes to provide a better understanding of gene function under external burden and provide more clues to engineer bacteria for biotechnological applications.

## Methods

### Bacterial strains, plasmids, and culture conditions

The *E. coli* B Strain *ER2566* was purchased from New England Biolabs (NEB). Cells were grown at 37 °C with sharking at 180 rpm in Luria-Bertani (LB) broth (5 g/L NaCl, 10 g/L tryptone, 5 g/L yeast extract, pH 7.0) under the pressure of continual passaging.

The gene encoding human papillomavirus 16 type L1 (HPV16L1) was cloned into the pTO-T7 expression vector. *E. coli* ER2566 with the pTO-T7-HPV16L1 vector was cultured in 250 mL LB broth containing 20 μg/mL kanamycin at 37 °C with shaking at 180 rpm. Upon reaching an OD_600_ of 1.0, 5 mL of culture was transferred into a flask containing 500 mL LB and 20 μg/mL kanamycin, and incubated at 37 °C with shaking at 180 rpm. At OD_600_ of 0.8, the culture was induced with a final concentration of 0.1 mM/L isopropyl-β-d-thiogalactopyranoside (IPTG) and incubated for 6 h at 37 °C with shaking at 180 rpm.

### DNA extraction and sequencing

Genomic DNA was extracted using a cetyltrimethylammonium bromide (CTAB)-based protocol [33]. Total genomic DNA concentration and quality were determined using a NanoDrop2000 Spectrophotometer (Thermo Fisher Scientific). DNA libraries for Illumina sequencing were constructed according to the manufacturer’s specifications (Thermo Fisher Scientific). After DNA library construction, sequencing was performed by a commercial service (Beijing Genomic Institute, Beijing, China) on an Illumina HiSeq2000 platform with 90-bp paired-end reads. Finally, 10.7 million raw reads were obtained for subsequent analyses.

### RNA extraction and sequencing

Cells were harvested by centrifugation at 7000 rpm for 10 min at room temperature and total RNA was extracted using the MasterPure RNA Purification Kit, according to the manufacturer’s protocol (Lucigen). DNase was added to reduce the chance of genomic DNA contamination. Total RNA was extracted in 50 μL RNase-free DEPC-treated water. RNA concentration was measured using an RNA Assay Kit in a Qubit 2.0 Fluorometer. A total of 3 μg RNA per sample was used as the input material for RNA sample preparations. Libraries of RNA-seq template were constructed using the NEBNext Ultra RNA Library Prep Kit for Illumina (NEB, USA), following the manufacturer’s recommendations. Sequencing was performed on an Illumina HiSeq 2500 platform and 125-bp paired-end reads were generated (Novogene, Beijing, China).

### Genome reannotation of the ER2566 strain

The ER2566 strain (NC_CP014268.2) whole genome was downloaded from the NCBI Reference Sequence Database as an input file for Prokka, a widely used annotation software that annotates a bacterial genome in about 10 min on a laptop [34]. To attain a rich and reliable annotation, we coordinated a suite of existing tools, including Prodigal 2.6 [35], Aragorn 1.2 [36], Barrnap 0.9 (https://github.com/tseemann/barrnap), Infernal 1.1 [37] and MinCED 0.2.0 [38] for our reannotation pipeline to predict, respectively, coding sequences (CDS), ribosomal RNA genes (rRNA), transfer RNA genes (tRNA), non-coding RNA genes (ncRNA) and clustered regularly interspaced short palindromic repeats (CRISPRs). Subsequently, various gene finders (GLIMMER 2.03 [39], GeneMark [40] and ZCURVE [41]) were used to further confirm the coding sequences.

### Variant calling of whole-genome sequencing by reads mapping

The quality of the raw reads were determined using FastQC [42], and appropriately truncated and filtered using fastp [43] to remove low-quality bases and Illumina adapter contamination with default parameters. The clean reads were then mapped against the C2566 reference genome using bwa-mem [44] with standard settings, and sorted by location as bam files. Bam files were converted to sam files using Samtools [45]. BactSNP was used to remove duplications and to detect variant calling [46].

### Variant calling of whole-genome sequencing by genome assembly

Draft contigs were created using SPAdes [47], with optimized parameters. The assembled draft contigs and sequencing libraries were used as input into Redundans [48] and scaffold genome assembly was performed with the recommended parameters. This resulted in fewer fragments, longer sequences and fewer gaps, as compared with using the input contigs. Scaffolds were then further improved using ABACAS [49], which rapidly aligned, ordered and orientated the scaffolds based on the following user-provided references: perl abacas.pl –r reference.fa –q scaffolds.fa –p nucmer. Finally, the assemblies were used to identify the mutants or indels against the reference genome through the program Harvest [50].

## Supplementary information

**Additional file 1: Table S1.** The list of the ruled-out genes in the reannotation.

**Additional file 2: Table S2.** The list of newly added protein-coding in the reannotation.

**Additional file 3: Table S3.** The list of complete CDSs in the reannotation.

**Additional file 4: Table S4.** Comparison with BL21(DE3) and ER2566 annotation.

**Additional file 5.** An integrated proteogenomics search database of ER2566 strain.

**Additional file 6: Table S5.** The list of newly added miscellaneous ncRNAs in the reannotation.

**Additional file 7: Table S6.** The transcription level of the newly added ncRNAs.

**Additional file 8: Table S7.** The top 50 the highly expressed genes.

**Additional file 9.** The re-sequenced ER2566 genome. (FASTA 4436 kb)

**Additional file 10.** The reannotation of ER2566 genome.

**Additional file 11: Figure S1.** The analysis pipeline for genomic re-annotation.

**Additional file 12: Figure S2.** The workflow of CDSs reannotation.

**Additional file 13: Figure S3.** Pairwise alignment and visualization. Assembly results for each step are assessed by alignment of final sequences back onto the reference genome. The visualization results are quickly achieved by *Nucmer*.

## Data Availability

The genomic sequence of the *E. coli* B strain ER2566 was downloaded in FASTA format from the NCBI-Microbial Genome Database (NZ_CP014268.2) and was annotated by Prokka using multiple annotation tools and manual review. Whole-genome re-sequencing and RNA-seq data in this study were respectively deposited in the NIH Sequence Read Archive (www.ncbi.nlm.nih.gov/sra/). The raw reads of whole-genome re-sequencing for different insert sizes:ER2566_500-SRR10828732 (https://trace.ncbi.nlm.nih.gov/Traces/sra/?run=SRR10828732), ER2566_2000-SRR10828731 (https://trace.ncbi.nlm.nih.gov/Traces/sra/?run=SRR10828731), RNA-Seq raw data:BB371-SRR10828730(https://trace.ncbi.nlm.nih.gov/Traces/sra/?run=SRR10828730), BB372-SRR10828729(https://trace.ncbi.nlm.nih.gov/Traces/sra/?run=SRR10828729), BB373-SRR108287828(https://trace.ncbi.nlm.nih.gov/Traces/sra/?run=SRR10828728), YY371-SRR108287827(https://trace.ncbi.nlm.nih.gov/Traces/sra/?run=SRR10828727), YY372-SRR108287826(https://trace.ncbi.nlm.nih.gov/Traces/sra/?run=SRR10828726), YY373-SRR108287825(https://trace.ncbi.nlm.nih.gov/Traces/sra/?run=SRR10828725). The assembly and reannotation of the ER2566 genome had been introduced a supplementary file (Additional files 9 and 10).

## Abbreviations

BLAST: Basic local alignment search tool
CDS: Coding sequence
IPTG: Isopropyl-β-d-thiogalactopyranoside
NGS: Next-generation sequencing
ORF: Open reading frame
PGAP: Prokaryotic genome annotation pipeline
RNA-seq: RNA sequencing
SNP: Single nucleotide polymorphism
WGS: Whole-genome sequencing
FPKM: Fragments per kilobase of transcript per million mapped reads
