# Supplementary material for: Genome re-sequencing and reannotation of the Escherichia coli ER2566 strain and transcriptome sequencing under overexpression conditions
Source: BMC Genomics. 2020 Jun 16;21:407. doi: 10.1186/s12864-020-06818-1 (PMC7296898; doi:10.1186/s12864-020-06818-1)
Supplement: Supplementary file 1 [file 12864_2020_6818_MOESM11_ESM.docx]

Fig S1.

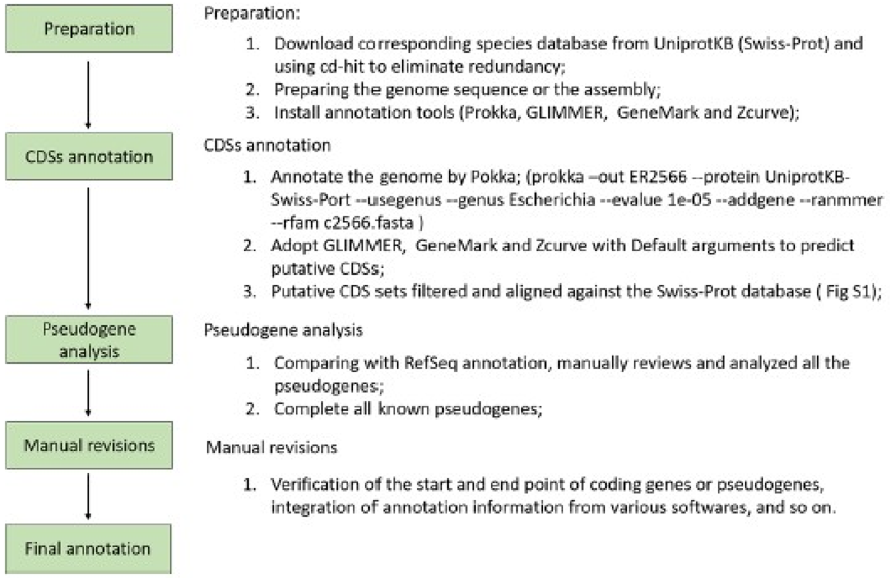
Preparation
CDSs annotation
Pseudogene analysis
Manual revisions
Final annotation
Preparation:
1. Download corresponding species database from UniprotKB (Swiss-Prot) and using cd-hit to eliminate redundancy;
2. Preparing the genome sequence or the assembly;
3. Install annotation tools (Prokka, GLIMMER, GeneMark and Zcurve);
CDSs annotation
1. Annotate the genome by Pokka; (prokka –out ER2566 --protein UniprotKB-Swiss-Port --usegenus --genus Escherichia --evalue 1e-05 --addgene --ranmmer --rfam c2566.fasta)
2. Adopt GLIMMER, GeneMark and Zcurve with Default arguments to predict putative CDSs;
3. Putative CDS sets filtered and aligned against the Swiss-Prot database (Fig S1);
Pseudogene analysis
1. Comparing with RefSeq annotation, manually reviews and analyzed all the pseudogenes;
2. Complete all known pseudogenes;
Manual revisions
1. Verification of the start and end point of coding genes or pseudogenes, integration of annotation information from various softwares, and so on.
