# Supplementary material for: Genome re-sequencing and reannotation of the Escherichia coli ER2566 strain and transcriptome sequencing under overexpression conditions
Source: BMC Genomics. 2020 Jun 16;21:407. doi: 10.1186/s12864-020-06818-1 (PMC7296898; doi:10.1186/s12864-020-06818-1)
Supplement: Supplementary file 2 [file 12864_2020_6818_MOESM12_ESM.docx]

Fig S2.

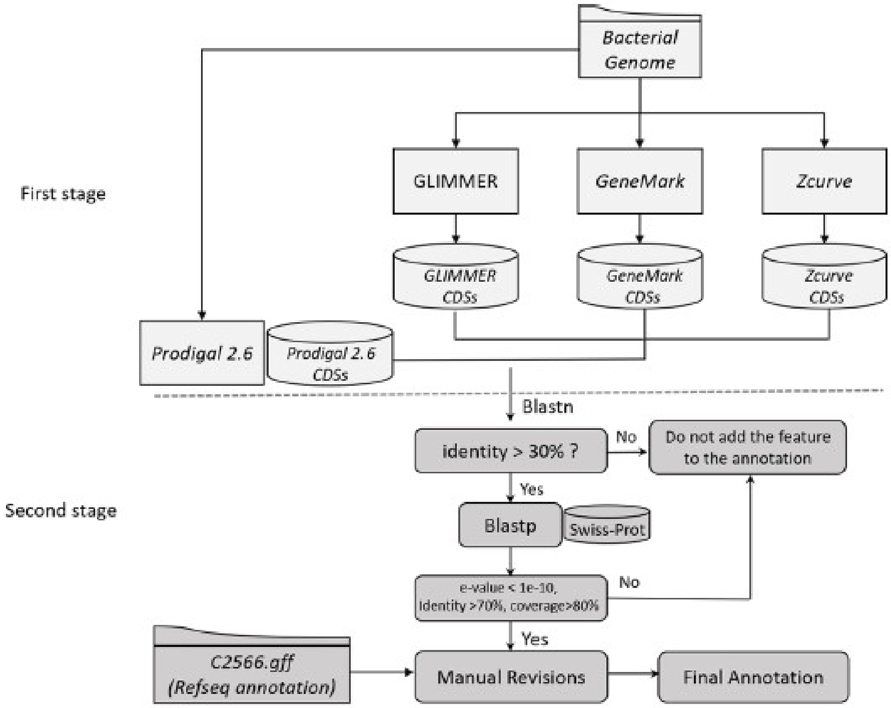

Bacterial Genome
First stage
GLIMMER
GeneMark
Zcurve
GLIMMER CDSs
GeneMark CDSs
Zcurve CDSs
Prodigal 2.6
Prodigal 2.6 CDSs
Blastn
identity > 30% ?
No
Do not add the feature to the annotation
Yes
Second stage
Blastp
Swiss-Prot
e-value < 1e-10, Identity >70%, coverage>80%
No
Yes
C2566.gff (Refseq annotation)
Manual Revisions
Final Annotation
