# Supplementary material for: Genome re-sequencing and reannotation of the Escherichia coli ER2566 strain and transcriptome sequencing under overexpression conditions
Source: BMC Genomics. 2020 Jun 16;21:407. doi: 10.1186/s12864-020-06818-1 (PMC7296898; doi:10.1186/s12864-020-06818-1)
Supplement: Supplementary file 3 [file 12864_2020_6818_MOESM13_ESM.docx]

Fig S3

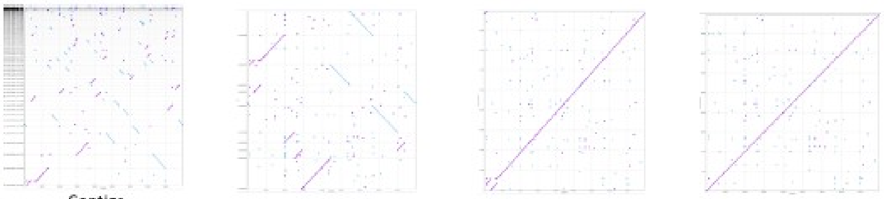

Contigs Scaffolds Assembly Re-sequence genome
